# Cardiovascular risk management following gestational diabetes and hypertensive disorders of pregnancy: a narrative review

**DOI:** 10.5694/mja2.51932

**Published:** 2023-05-07

**Authors:** Simone Marschner, Anushriya Pant, Amanda Henry, Louise J Maple‐Brown, Lisa Moran, N Wah Cheung, Clara K Chow, Sarah Zaman

**Affiliations:** ^1^ Westmead Applied Research Centre University of Sydney Sydney NSW; ^2^ University of New South Wales Sydney NSW; ^3^ St George Hospital Sydney NSW; ^4^ Diabetes across the Lifecourse: Northern Australia Partnership, Menzies School of Health Research Darwin NT; ^5^ Royal Darwin Hospital Darwin NT; ^6^ Monash Centre for Health Research and Implementation Monash University Melbourne VIC; ^7^ Monash Health Melbourne VIC; ^8^ Westmead Hospital Sydney NSW

**Keywords:** Diabetes, gestational, Pregnancy complications, Hypertension, Diabetes mellitus, type 2

**Summary**

- Gestational diabetes mellitus and hypertensive disorders of pregnancy (gestational hypertension and preeclampsia) are strong independent risk predictors for future cardiovascular disease (CVD) specific to women.
- Awareness of the relationship between pregnancy‐related risk factors and CVD needs improvement among both women and clinicians.
- Education of patients and their health care providers is urgently needed to ensure preventive measures are implemented across a woman's lifespan to care for the health of women affected by these conditions.
- Few interventions have been developed or studied which are designed to lower CVD risk in women with pregnancy‐related risk factors.
- Future work should focus on developing interventions that are tailored together with individual communities and integrated within health care systems, ensuring each health care provider's role is clearly outlined to effectively prevent and manage CVD in these high risk women.

Gestational diabetes mellitus rates have tripled in Australia in the past 20 years, with 16.7% of women (age standardised, > 44 000 women) who gave birth in hospital in 2019–2020 diagnosed with gestational diabetes, compared with 5.2% in 2000–2001.^1^, ^2^ This is an alarming upward trend even after allowing for changes in the diagnostic criteria for gestational diabetes, with the International Association of Diabetes and Pregnancy Study Groups (IADPSG) criteria being accepted by the Australasian Diabetes in Pregnancy Society in 2014.^3^ Similar trends are observed globally. In the United States, gestational diabetes was diagnosed in 7.8 per 100 births, an increase of 30% from 2016.^4^ Hypertensive disorders of pregnancy (HDP), including de novo gestational hypertension and preeclampsia, affected 3.4% (about 7500 women) of Australian pregnant women in 2020.^2^ Although HDP rates appear relatively stable in Australia,^2^ it affects 10.7% of pregnant women in the US, where rates are increasing.^5^ In parallel, Australian women today have a high burden of cardiovascular disease (CVD) and risk factors; 31.4% have hypertension,^6^ 3.8% (age standardised) have known diabetes mellitus,^7^ 4.8% of women have CVD,^8^ the leading cause of death for women in Australia.^8^ Taken together, these statistics show a growing cardiovascular health problem for Australian women. However, they also highlight a unique opportunity for CVD screening and prevention specific to women through a better understanding and management of the link between pregnancy‐related risk factors and CVD.

This narrative review synthesises the literature on gestational diabetes, hypertension and their relationship with future cardiovascular risk, as well as screening and intervention strategies to mitigate this risk. For this review, we searched the PubMed (via Medline) online database for systematic reviews published between January 2019 and October 2022.

## Gestational diabetes

### Burden and trends

Unlike type 1 diabetes, gestational diabetes is not caused by a lack of insulin but by other hormones produced during pregnancy that can make insulin less effective. The inability to compensate results in relative insulin resistance, which resolves following delivery. The increase of gestational diabetes in Australia is consistent with other high income regions of North America and began before, and continued past, the changed diagnostic criteria (since 2013)^1^, ^4^ and may be due to rising obesity rates and higher maternal age. From 2014 to 2017–2018, the proportion of mothers who are obese has risen from 20% to 23%, and the proportion of women giving birth aged over 35 years has risen from 22% to 26% (Box 1).^1^, ^2^ Gestational diabetes rates are even higher in some ethnicities, including South East Asian (1.7 times), and North African, Middle Eastern and North East Asian women (1.6 times).^1^ One study in Australia found women born in South Asia had an odds ratio (OR) of 4.33 for gestational diabetes (95% CI, 4.12–4.55), relative to women born in Australia.^9^ First Nations Australian women experience a 1.3‐fold higher rate of gestational diabetes compared with non‐Indigenous women.^10^

Box 1 **Trends in risk factors in Australian pregnant women**

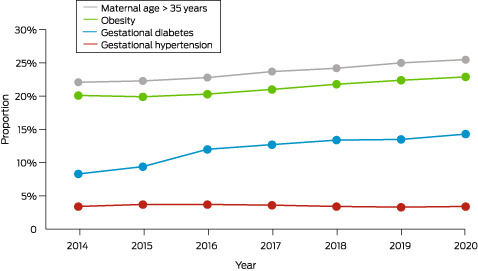

### Type 2 diabetes

Gestational diabetes is strongly associated with future type 2 diabetes. Several meta‐analyses have found that women with gestational diabetes have a six‐ to tenfold risk of developing type 2 diabetes.^11^, ^12^, ^13^, ^14^ It is estimated that 10–31% of parous women with type 2 diabetes would have experienced a gestational diabetes pregnancy earlier.^14^ The incidence rate of type 2 diabetes after gestational diabetes increases by about 10% with every ten years after the gestational diabetes diagnosis.^15^ The risk seems higher in South Asian women, with an 11‐fold risk of subsequent type 2 diabetes and a cumulative incidence of 17% at five years and 33% at ten years.^16^ Smaller studies among Aboriginal and Torres Strait Islander women also indicate increased risk; for example, the Pregnancy and Neonatal Diabetes Outcomes in Remote Australia (PANDORA) study found that 13% of First Nations women with gestational diabetes developed type 2 diabetes (*n* = 11/82), compared with none of the non‐Indigenous women with gestational diabetes (*n* = 0/92 women), after a median follow‐up of only 2.5 years.^17^

### Cardiovascular disease

Women with gestational diabetes have higher levels of traditional cardiovascular risk factors. A meta‐analysis showed higher systolic blood pressure (mean difference, 2.47 mmHg; 95% CI, 1.74–3.40 mmHg), body mass index (mean difference, 1.54 kg/m^2^, 95% CI, 1.32–2.46 kg/m^2^), low‐density lipoprotein cholesterol (standardised mean difference [SMD], 0.19; 95% CI, 0.08–0.30), triglycerides (SMD, 0.56; 95% CI, 0.42–0.70), and glucose (SMD, 0.69; 95% CI, 0.56–0.81), and lower high‐density lipoprotein cholesterol (SMD, −0.28; 95% CI, −0.39 to −0.16)^18^ developing as early as one year post partum.^18^ Together, these changes equate to a threefold increased risk of metabolic syndrome in women with gestational diabetes.^19^

It is not surprising then that women with previous gestational diabetes have a twofold risk of CVD events (relative risk [RR], 1.98; 95% CI, 1.57–2.50) with the RR being greater in the first decade (RR, 2.31; 95% CI, 1.57–3.39).^20^ Similarly, women with gestational diabetes had increased risk of coronary artery disease (RR, 1.40; 95% CI, 1.18–1.65), myocardial infarction (RR, 1.74; 95% CI, 1.37–2.20), heart failure (RR, 1.62; 95% CI, 1.29–2.05) and stroke (RR, 1.45; 95% CI; 1.29–1.63).^21^ Importantly, the risk for CVD in these women occurred irrespective of the development of traditional risk factors and was consistent across time.^20^, ^21^ However, many of the studies assessed the risk of women diagnosed with gestational diabetes before the criteria change in 2014. The above data may therefore overestimate the risk for the current cohort which includes milder cases of gestational diabetes.

## Hypertensive disorders of pregnancy

### Burden and trends

HDP encompass chronic hypertension, gestational hypertension (new onset of high blood pressure without proteinuria during pregnancy), preeclampsia and/or eclampsia, and preeclampsia superimposed on chronic hypertension. Although the rate of HDP in Australia has remained relatively stable (Box 1), HDP prevalence appears higher in Australia (5.7–8.2% for gestational hypertension and 2.6–9.2% for preeclampsia) compared with Europe (0.9–5.8% and 1.6–5.2% respectively), although similar to North America (1.5–4.0% and 3.0–8.0% respectively).^22^ However, in the US, the rates of overall HDP have increased from 8.9% in 2010 to 14.9% in 2019.^5^ In First Nations Australian women, the risk of pregnancy‐related hypertension has been reported as 66% greater than in non‐Indigenous women.^23^

### Cardiovascular disease

Women with prior HDP are at a threefold higher risk of hypertension (RR, 3.46; 95% CI, 2.67–4.49), which is higher in the first five years post partum (RR, 5.34; 95% CI; 2.74–10.39).^24^ A large Danish study (*N* = 482 972) estimated that 14% of women who had HDP in their 20s developed hypertension within a decade and 32% within two decades, suggesting women with HDP are frequently developing hypertension in their 30s and 40s.^25^ The risk was similar for the first year for gestational hypertension and severe preeclampsia, but longer term risk was significantly higher for women with gestational hypertension.^25^ For preeclampsia, the risk is higher among patients with early onset preeclampsia.^26^

The cardiovascular risk for women with HDP extends well beyond hypertension, with a twofold higher risk of developing type 2 diabetes,^27^ even after adjusting for coexisting gestational diabetes (hazard ratio [HR], 2.01; 95% CI, 1.77–2.28).^28^ Women diagnosed with HDP have a higher risk of cardiovascular death (OR, 2.18; 95% CI, 1.8–2.7) and major cardiovascular events (OR, 1.80; 95% CI, 1.6–2.0).^27^ Looking at the components of HDP, women with gestational hypertension have a twofold increased risk of CVD and a 1.8 risk of heart failure,^29^ and women with preeclampsia have a threefold increased risk of premature‐onset coronary artery disease.^30^ For women with preeclampsia, coronary heart disease, heart failure and stroke were substantially higher in the first one to ten years after an affected pregnancy.^29^ The risk can also be immediate in women with HDP, with an about twofold increase in severe cardiovascular outcomes, such as myocardial infarction and stroke, from pregnancy through to 60 days after birth.^31^

Heart failure with preserved ejection fraction (HFpEF)^32^ is an emerging global health problem with a female predominance and often associated with hypertension.^33^ A retrospective cohort study using the New York and Florida Inpatient Databases (2006–2014) found a twofold increased risk of HFpEF hospitalisation among women with a history of preeclampsia or eclampsia. The median time from pregnancy to heart failure was short, at only 32 months, and women affected were young (median age, 34 years).^34^

### Chronic kidney disease

A recent Swedish registry‐based study showed a high risk of developing chronic kidney disease after preeclampsia (HR, 1.92; 95% CI, 1.83–2.03).^35^ This was further supported by a multicentre study in France, where the prevalence of newly diagnosed chronic kidney disease was high after preeclampsia (19% *v* expected 3% in women of childbearing age).^36^

## Pathophysiology

Pregnancy is a complex interaction between the maternal and fetal environments, with physiological changes that stress a woman's body to adapt and sustain the energy demands of the fetus. What remains unclear is the exact pathophysiology of elevated and premature CVD risk in women with gestational diabetes and HDP. The first hypothesis is that women enter the pregnancy at elevated cardiometabolic risk either from genetic or environmental predisposition. This is supported by the finding that patients with gestational diabetes, in particular, have evidence of pre‐pregnancy cardiometabolic changes, such as higher body mass index, dyslipidaemia, and abnormal diabetic markers.^18^ The second hypothesis is that the pregnancy condition itself is a mechanistic driver of premature CVD due to abnormal placentation, inflammation, and endothelial dysfunction.^37^

In women with gestational diabetes, the pancreatic β‐cells fail to compensate for placental‐mediated insulin resistance, which leads to hyperglycaemia.^38^ The risk of developing type 2 diabetes for women with gestational diabetes may be due to progressive impairment of β‐cells and insulin resistance.^38^ Although the relationship between CVD and pregnancy‐related diabetes is poorly understood, it can be hypothesised that this hyperglycaemic state increases the release of inflammatory cytokines that promote oxidative stress and atherogenesis, both drivers for CVD development.^39^

Preeclampsia has been associated with impaired placentation, disrupted maternal haemodynamics and endothelial dysfunction with subsequent maternal end‐organ damage.^40^ In women with HDP, there is defective placentation causing inadequate uterine placental blood flow, which results in a hypoxic state known as placental ischaemia.^40^, ^41^ Here, women with preeclampsia have enhanced expression of modulators of angiogenesis, inflammatory cytokines, and oxidative stress, believed to lead to endothelial dysfunction.^41^ This endothelial dysfunction is a systemic pathological state that progresses to atherosclerosis, likely contributing to premature coronary artery disease and cardiovascular events.^30^, ^42^ In addition, gestational hypertension can result in arterial stiffness, another factor in the development of premature atherosclerosis and CVD.^40^

## Cardiovascular risk reduction after pregnancy

### Early detection

Screening is important in early detection of cardiovascular risk factors, including early type 2 diabetes diagnosis, with evidence that post partum testing is suboptimal.^43^ Recommendations from the Royal Australian College of General Practitioners, consistent with international guidelines, urge women with gestational diabetes to have follow‐up screening with a glucose tolerance test six to 12 weeks post partum and every one to three years thereafter.^44^, ^45^ Although less standardised, both national and international guidelines recommend that women with HDP have post partum follow‐up assessment of cardiovascular risk factors and counselling regarding healthy lifestyle to both reduce HDP recurrence in subsequent pregnancies and decrease ongoing cardiometabolic risk.^46^, ^47^ The American College of Obstetricians and Gynecologists recommends women with HDP undergo blood pressure screening seven to ten days after delivery, yet a study in Atlanta (*N* = 1260) found 13.7% attended a blood pressure screening visit within ten days of delivery.^48^ Contacting women, even within a short time after the pregnancy, is difficult. Systematic reviews show screening rates less than 58% at four months post partum, with little improvement in the past ten years.^49^ Identified barriers to post partum screening include:

- difficulties in handover between primary and secondary care (ambiguous roles and communication difficulties);
- short term focus in clinical consultations (underplaying the risk so as not to overwhelm women and competing priorities with a new baby); and
- patient‐centric barriers such as time pressures.^50^

Reminder systems are very helpful. The GooD4Mum reminder system conducted in Australia resulted in over a doubling of the proportion of women with gestational diabetes that were screened in the first year.^51^ However, a pilot study of post partum reminder messages in the remote Northern Territory reported challenges contacting and/or engaging women and that successful messaging was not associated with higher rates of any post partum blood glucose testing.^52^

### Interventions targeting women with gestational diabetes and HDP

A systematic review of randomised controlled trials (RCTs) on women with previous gestational diabetes showed that early (within 3 years) lifestyle interventions on diet and physical activity were effective in reducing the risk of post partum type 2 diabetes (RR, 0.57; 95% CI, 0.42–0.78).^53^ Another meta‐analysis showed a risk reduction of 25% with the results more effective in trials offering intervention soon after delivery (less than six months post partum).^54^ However, a more recent large RCT found that a 12‐month lifestyle intervention in South Asian women with gestational diabetes did not prevent subsequent glycaemic deterioration.^55^ A systematic review on women with gestational diabetes explored lifestyle interventions and screening programs, finding that participation in screening rose to 40%, but a woman's knowledge of their risk of developing future type 2 diabetes was still low.^56^ Encouragement of breastfeeding is an important way to lower risk, with two separate systematic reviews finding an RR of 0.73 (95% CI, 0.65–0.83)^57^ and 0.66 (95% CI, 0.48–0.90)^58^ for women who breastfed for any duration versus women who did not breastfeed, and a 1% lower risk of developing type 2 diabetes for every additional month of breastfeeding after birth.^57^ Innovative lifestyle interventions in women with gestational diabetes are ongoing; for example, wearing ankle weights during routine daily activities such as cleaning or childcare^59^ or using digital health technology to promote activity and education (Box 2 and Box 3).

Box 2 **Studies assessing interventions to reduce cardiovascular disease (CVD) risk in women with gestational diabetes mellitus (GDM) and women with hypertensive disorders of pregnancy (HDP)**

**Table:** 

Study author, year (study name)	Study design	Population	Intervention	Comparator	Primary outcomes	Intervention *v* comparator	Limitations
**GDM**							
Tandon et al, 2022 (LIVING)^55^	• RCT • 12 months • South Asia*	• *N* = 1601 • GDM	• Lifestyle interventions: ➤ diet, PA ➤ voice/text messages	• Usual care	• Glycaemia	• Negative study result (HR, 0.92; 95% CI, 0.76–1.12; *P* = 0.42)	• 19.8% had the primary outcome missing; COVID‐19 lockdowns meant 48.9% received some intervention remotely • May reduce effect size
Lim et al, 2021 (SPAROW)^60^	• RCT • 4 months • Singapore	• *N* = 200 • GDM	• App logging weight, meals, PA; web‐based specialist chat	• Standard care	• Weight	• Negative study result (OR, 1.40; 95% CI, 0.76–2.58)	• High retention, but at 4 months, participant engagement with the intervention was 60.8% (SD, 33.9%) • May reduce effect size
Cheung et al, 2019 (SmartMums)^61^	• RCT (pilot) • 6 months • Australia	• *N* = 60 women with GDM	• Text messaging; activity monitor; face‐to‐face diet counselling	• Paper‐based information	• GTT by 12 weeks post partum • Weight (kg)	• Negative study result • Intervention: 28/40 (70%) • Control: 13/20 (65%; *P* = 0.77) • Intervention: −1.7 ± 4.1 • Control: −1.1 ± 3.3 (*P* = 0.47)	• Small study
Li et al, 2020^15^, ^53^	• Systematic review (15 studies) • 10.2 weeks to 3 years	• *N* = 43–2280 • RCT • GDM • Lifestyle	• Lifestyle intervention: diet, PA	• Usual care	• Type 2 diabetes mellitus	• Positive meta‐analysis • 10 studies intervention after pregnancy (RR, 0.57; 95% CI, 0.42–0.78)	• Lifestyle intervention within 3 years of GDM diagnosis
Goveia et al, 2018^54^	• Systematic review (14 studies)	• *N* = 43–450 • RCT, GDM, lifestyle intervention	• Lifestyle intervention: diet, PA, breastfeeding	• Standard care	• Type 2 diabetes mellitus	• Negative and positive meta‐analysis • 8 studies with type 2 diabetes mellitus outcome (RR, 0.75; 95% CI, 0.55–1.03) • 5 studies with type 2 diabetes mellitus (intervention < 1 year; RR, 0.61; 95% CI, 0.40–0.94)	
Ferrara et al, 2016 (GEM)^62^	• RCT • 6 months • United States	• *N* = 2280 • GDM	• Mail: weight goal • Phone sessions: coach and dietician	• Usual care	• Weight	• Positive study result (OR, 1.28; 95% CI, 1.10–1.47)	• Sound cluster RCT • Balanced by treatment group, but 26.5% of eligible women did not participate
Ratner et al, 2008 (DPP)^63^	• RCT • 3 years • United States	• *N* = 350 • GDM	• Metformin • Intensive lifestyle program	• Placebo	• Type 2 diabetes mellitus	• Positive study result • Reduce type 2 diabetes mellitus by 50.4% and 53.4% compared with placebo	
**HDP**							
Riemer et al, 2021^64^	• RCT • 6 months • Germany	• *N* = 38 • Severe HDP	• Nutritional advice, Mediterranean diet • Exercise program	• Control	• Aortic pulse wave velocity	• Negative study result • Intervention (6.36 ± 0.76 m/s) *v* control (7.33 ± 2.25 m/s)	• Small study
Hutchesson et al, 2020^65^	• RCT (pilot) • 3 months • Australia	• *N* = 31 preeclampsia	• Web‐based lifestyle intervention and weekly email newsletters	• Links: National Heart Foundation of Australia website^†^	• Program acceptability	• Overall acceptability high (84.6% satisfied)	• Small pilot study
Lui et al, 2019^66^	• Systematic review (2 studies)	• *N* = 151–201 preeclampsia	• 500 mg calcium every day • Web‐based specialist education	• Placebo	• Blood pressure • Diet and PA	• Positive study result • Healthy diet (*P* = 0.03) • Knowledge of CVD risk (*P* = 0.01), less physical inactivity (*P* = 0.0006) • No effect on blood pressure	• Women < 10 years post partum after HDP

App = smartphone application; COVID‐19 = coronavirus disease 2019; DPP = Diabetes Prevention Program; GEM = Gestation Diabetes Effects on Moms; GTT = glucose tolerance test; HR = hazard ratio; LIVING = Lifestyle Intervention in Gestational Diabetes; OR = odds ratio; PA = physical activity; RCT = randomised controlled trial; RR = relative risk; SD = standard deviation; SPAROW = Smartphone App to Restore Optimal Weight. * India, Sri Lanka, Bangladesh. † National Heart Foundation website: www.heartfoundation.org.au.

Box 3 **Future/ongoing studies targeting women with gestational diabetes mellitus (GDM) and women with hypertensive disorders of pregnancy (HDP) to reduce cardiovascular disease (CVD) risk**

**Table:** 

Study author, year (study name)	Study design	Population	Intervention	Comparator	Outcome
Nielsen et al, 2020 (Face‐it study)^67^	• RCT • 12 months • Denmark	• *N* = 460 • GDM	• Additional home health care visits • Digital application: health coaching • Face‐to‐face counselling	• Usual care	• BMI
Stith et al, 2021 (Moms in motion)^59^	• RCT • 6 months • United States	• *N* = 160 • GDM	• 1 kg ankle weights during routine activities	• Usual care	• Weight
Marschner et al, 2021 (SmartMums2)^68^	• RCT • 12 months • Australia	• *N* = 180 • GDM	• Usual care, activity monitor, and customised text messaging	• Usual care with activity monitor	• Healthy lifestyle, must meet two of the three criteria: weight, PA, diet
CAC‐Women Trial, 2022^69^	• RCT • 12 months • Australia	• *N* = 700 • GDM • HDP	• Computed tomography coronary artery calcium score‐guided approach	• Delayed calcium score	• Systolic BP (mmHg) and LDL‐C (mmol/L)
Henry et al, 2020 (BP^2^)^70^	• RCT • 12 months • Australia	• *N* = 480 • HDP	• Lifestyle counselling on CVD risk profile, exercise, and healthy diet with obstetrician, physician and dietitian • Telephone‐based lifestyle program delivered by dieticians and exercise physiologists	• Usual care	• BP • Weight • Waist circumference
HH4NM study, 2022 (ClinicalTrials.gov, NCT03749746)	• RCT • 12 months • United States	• *N* = 148 • Obesity and preeclampsia	• Web‐based lifestyle, home BP monitor	• Usual care	• Weight

BMI = body mass index; BP = blood pressure; CAC = coronary artery calcium; HH4NM = Heart Health 4 New Moms; LDL‐C = low‐density lipoprotein cholesterol; PA = physical activity.

Interventions targeting women with HDP are even more scarce. A 2019 systematic review found only two RCTs with no evidence of improvement.^66^ More recently, small pilot studies have shown feasibility and acceptability of web‐based CVD prevention and physical exercise interventions,^64^, ^65^ but require confirmation in larger studies. Box 2 details these studies^64^, ^65^, ^66^ and Box 3 highlights other ongoing larger RCTs,^70^ including the innovative multisite CAC‐Women Trial (*N* = 700)^69^ currently underway which will target women with at least one risk‐enhancing factor of HDP, gestational diabetes and/or premature menopause. Computed tomography coronary artery calcium scoring will identify women with premature subclinical atherosclerosis and guide risk factor counselling.

### Barriers to interventions in women with gestational diabetes and HDP

The post partum period is a challenging time to adopt a healthy lifestyle, and the busy life of a mother can prevent adequate screening. Qualitative studies have explored the barriers to interventions for women with gestational diabetes and HDP and identified barriers such as the role as a mother, lack of social support, demands of life, personal preferences and experiences, risk perception and information, and limited finances and resources.^71^ Addressing knowledge, risk perception, fear of type 2 diabetes diagnosis, low prioritisation of personal health, and fatalism have been found to be key factors affecting post partum type 2 diabetes screening.^72^ One study in women with HDP of a computer‐tailored health education program had only 23% compliance, with the cited major barrier being lack of time.^73^ Another critical barrier is education, with women often found to have limited or no knowledge about the link between HDP and CVD.^74^ Education has also been found to be a barrier for First Nations women with gestational diabetes, for whom changes to social and structural determinants of health are required to address these gaps.^75^ A further barrier which interventions need to combat, particularly in Australia, is the vast remoteness of some communities and the inequities related to the social determinants of health, especially among First Nations women.^76^ Interventions must be designed in partnership with women and communities and adapted locally for different contexts and populations. The Aboriginal and Torres Strait Islander Advisory Group of the Diabetes across the Lifecourse: Northern Australia Partnership is actively involved in the codesign of a suite of interventions to reduce diabetes‐related risk at key time points in a woman's life course, including the post partum period (https://diabeteslifecourse.org.au/). System and structural level change, in partnership with First Nations communities, is urgently required to address the social determinants of health, including poverty, education, food security, employment and housing.

### Implementation of interventions following gestational diabetes and HDP

The diagnosis of gestational diabetes or HDP can be confronting and come as a shock to a pregnant woman and requires education and professional support.^77^ As well as supporting women through the diagnosis and pregnancy, clinicians need to recognise that these diagnoses are significant when evaluating a woman's risk of serious future CVD outcomes. Discussion and screening for cardiovascular risk factors and disease needs to be routine and embedded in primary care, linked to obstetric and other specialist services. Qualitative studies show that women want risk counselling and more structured postnatal support with automated reminders.^78^, ^79^ Evidence‐based lifestyle changes should be encouraged and supported, and education of their elevated risk is required to motivate women to adhere to lifestyle changes. Using the unique window of a diagnosis of gestational diabetes or HDP to identify high risk women, Box 4 illustrates an intervention pathway to reduce the burden of CVD. We need to raise awareness of the risks and optimise post partum management of high risk women through structured assessment, care and tailored interventions.

Box 4 **Improving cardiovascular outcomes after gestational diabetes and hypertensive disorders of pregnancy**

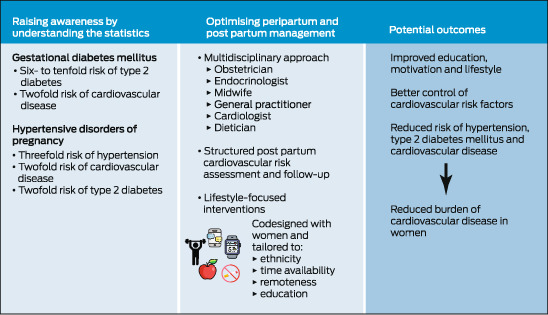

The American Heart Association has declared a call to action, as pregnancy‐related risks in women have been ignored for too long.^80^ However, we still have a long way to go in practice and policy to incorporate gestational complications into CVD risk calculators, as well as to guide clinicians on appropriate medical therapy to lower CVD risk.

## Conclusion

Pregnancy can be seen as a stress test for cardiometabolic conditions, where the physiological demands of pregnancy can unmask women at risk for CVD as well as predispose to pathophysiological changes that lead to premature atherosclerosis. Gestational diabetes and HDP increase the risk of CVD, and this risk occurs in both early and late post partum. However, awareness of this risk among women and health care providers is low. Interventions are currently being developed and tested, largely focused on improving screening, promoting lifestyle changes, and finding ways to detect early onset CVD. However, much of this research is in its infancy and it will be many years before translation into clinical practice guidelines occurs. What remains clear is that it is imperative that clinicians educate themselves and their patients on the elevated CVD risk seen following gestational diabetes and HDP. Screening for, and treatment of, cardiovascular risk factors and disease needs to start soon after an affected pregnancy and continue throughout a woman's life course.

## Open access

Open access publishing facilitated by The University of Sydney, as part of the Wiley ‐ The University of Sydney agreement via the Council of Australian University Librarians.

## Competing interests

No relevant disclosures.

## Provenance

Commissioned; externally peer reviewed.
